# Metaproteomic and Metagenomic-Coupled Approach to Investigate Microbial Response to Electrochemical Conditions in Microbial Fuel Cells

**DOI:** 10.3390/microorganisms11112695

**Published:** 2023-11-03

**Authors:** Alexiane Godain, Timothy M. Vogel, Jean-Michel Monnier, Agathe Paitier, Naoufel Haddour

**Affiliations:** 1 Ecole Centrale de Lyon, INSA Lyon, University Lyon, Université Claude Bernard Lyon 1, CNRS, Ampère, UMR5005, 69130 Ecully, France; 2 Laboratoire d’Ecologie Microbienne, Universite Claude Bernard Lyon 1, UMR CNRS 5557, UMR INRAE 1418, VetAgro Sup, 69622 Villeurbanne, France

**Keywords:** microbial fuel cell, metagenomic, metaproteomic, electroactive bacteria, extracellular electron transfer

## Abstract

MFCs represent a promising sustainable biotechnology that enables the direct conversion of organic matter from wastewater into electricity using bacterial biofilms as biocatalysts. A crucial aspect of MFCs is how electroactive bacteria (EAB) behave and their associated mechanisms during extracellular electron transfer to the anode. A critical phase in the MFC start-up process is the initial colonization of the anode by EAB. Two MFCs were operated with an external resistance of 1000 ohms, one with an applied electrical voltage of 500 mV during the initial four days of biofilm formation and the other without any additional applied voltage. After stabilization of electricity production, total DNA and protein were extracted and sequenced from both setups. The combined metaproteomic/metagenomic analysis revealed that the application of voltage during the colonization step predominantly increased direct electron transfer via cytochrome c, mediated primarily by *Geobacter* sp. Conversely, the absence of applied voltage during colonization resulted in a broader diversity of bacteria, including *Pseudomonas* and *Aeromonas*, which participated in electricity production via mediated electron transfer involving flavin family members.

## 1. Introduction

Microbial fuel cells (MFCs) serve as a sustainable biotechnological solution, facilitating the direct conversion of organic waste into electricity through bacterial biofilms as biocatalysts [1,2,3,4]. Electroactive bacteria (EAB) play a pivotal role in this process as they can interact electrically with each other and/or their extracellular environments, leading to the oxidation of organic matter and electron transfer to an anode. Over the past decade, advancements in MFC technology have led to an increase in reported power densities [5,6]. A critical phase in the MFC start-up process is the initial colonization of the anode by EAB. This phase influences the bacterial community structure of the mature biofilm and subsequently affects the overall electron transfer rate. To optimize this process, a comprehensive understanding of the EAB community and their electron transfer mechanisms is essential. Various bacteria employ redox shuttles, such as Flavin-type molecules in *Shewanella* or phenazine-type in *Pseudomonas* [7]. Another prevalent mechanism involves direct electron transfer through c-type cytochrome, as observed in species like *Clostridium*, *Geobacter*, and *Shewanella* [8,9]. Several strategies have been proposed to enhance the growth of EAB on the anode. For instance, the introduction of different substrates has been explored to determine if specific bacteria could be selectively promoted on the anode. Studies have indicated that acetate addition promotes the growth of the acetotroph bacterium *Geobacter*, which is electroactive. This led to an enhanced electricity production [10,11]. However, the chemical nature of the electron donor might not be the sole determinant influencing bacterial selection in MFCs. Electrical and electrochemical parameters, such as the electrochemical anodic potential and external resistance, have been proposed to play significant roles in determining the type and level of anodic respiration [3,12,13,14,15]. Wang et al. demonstrated that applying an anode potential of 200 mV (vs. Ag/AgCl) could expedite the MFC start-up, although a comprehensive bacterial community analysis was not conducted [14]. Furthermore, Wei et al. indicated that a positive anode potential (vs. Ag/AgCl) reduced the start-up duration and increased the current generation for *G. sulfurreducens* [15]. Conversely, Aelterman et al. did not observe that a negative anode potential (−200 mV vs. Ag/AgCl), which enhanced current generation, affected the start-up duration [12]. Torres et al. observed that a negative anode potential favored the selection of *Geobacter*, while a positive anode potential resulted in a more diverse bacterial community [13]. The external resistance has also been identified as a significant factor influencing both electrical performance and bacterial community selection. Studies by Aelterman et al. [16] and Zhang et al. [17] posited that the optimal external resistance should be minimal, yet proximate to the internal resistance, to achieve superior power density. Additionally, Godain et al. noted that variations in external resistance could lead to distinct bacterial communities on the anode [3]. While the effects of electrochemical anodic potential and external resistance on MFCs have been extensively researched, other parameters, such as the potential difference between the anode and cathode, remain under-explored. This potential difference could exert an influence on the anodic bacterial community within the MFC by generating an electromotive force during the start-up phase. This electromotive force could potentially facilitate the migration of microorganisms to the anode, thereby altering the taxonomic and functional attributes of the anodic biofilm. Upon the application of an external voltage, a selective pressure is exerted on the microbial community, which favors taxa and metabolic functions adept at interfacing with the electrode. Such observations are corroborated by studies like that of Yang et al., who investigated the bioelectrochemical enhancement of the anammox process for nitrogen removal. Their study indicated that voltage application augmented the abundance of denitrifying functional groups [18]. In bioelectrochemical systems, the applied voltage has been shown to modulate the synthesis of specific proteins, predominantly those implicated in extracellular electron transfer mechanisms. These proteins play a pivotal role in microbial-electrode interactions, leading to their heightened representation in the community proteome dynamics under voltage-induced conditions. A distinct study by Zhang et al. examined the effect of voltage application on the biodegradation of certain pharmaceuticals in a bioelectrochemical reactor. Their observations elucidated that the application of voltage facilitated the attenuation of certain drugs, indicating the influence of voltage on microbial activity [19]. Moreover, in environments subjected to applied voltage, specific functions and pathways directly associated with electron transfer are favored, leading to the manifestation of a specialized set of proteins. For instance, Carrillo-Peña et al. explored the bioelectrochemical enhancement of methane production from an exhausted vine shoot fermentation broth [20]. Their findings revealed that microbial electrolysis cells under applied voltage were colonized predominantly by the genus *Methanosarcina*, thereby promoting methanogenesis via both acetoclastic and hydrogenotrophic pathways. This not only facilitated enhanced carbon utilization but also elevated biogas production relative to the unpolarized cell. The bacterial community in both cells was similar but a clear adaptation of *Methanosarcina* Archaea was exhibited in the polarized cell, which could explain the increased yields in CH_4_ production. Recent studies have delved into the taxonomic diversity and structure of mixed-species anodic biofilms of MFCs [4,21,22,23,24,25,26,27]. These studies have highlighted the variability in major taxa across different studies with Deltaproteobacteria [26,27], Betaproteobacteria [21], or Bacteroidetes [24] being observed as predominant bacteria under different conditions. The relationship between diversity parameters and power production has also been explored with some studies indicating a positive correlation between the Shannon index and power production [25,26]. One of the few studies that delved into the potential functional capabilities of microorganisms in the anodic biofilm utilized metagenomic data by annotating sequences to genes known for enzymes potentially involved in extracellular electron transfer [28]. However, these genes only signify microbial potential and not their activity. Metaproteomics has been recognized as a promising method to access bacterial ecosystem function [29,30]. Leary et al. used metaproteomics to investigate the impact of the cathodic electrochemical potential on gene expression in the cathodic biofilm [31]. A combined metaproteomics and metagenomics approach would offer a more comprehensive insight into both aspects. To the best of our knowledge, no studies have applied metaproteomics to anodic biofilms. This study explored the influence of an applied voltage between the anode and cathode on the bacterial community structure and activity, employing a novel combined metaproteomics/metagenomics methodology. This integrative approach not only offers a comprehensive insight into community structure but also unravels the intricate interplay of microbial activity in response to applied voltage.

## 2. Materials and Methods

### 2.1. MFC Setup

**Figure 1:**
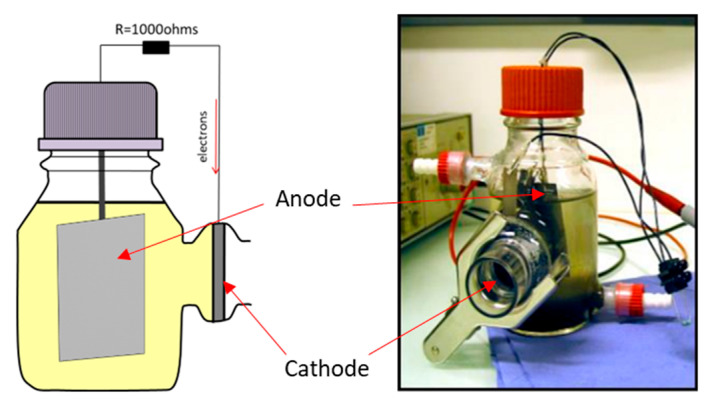
Single-chamber batch MFC with air cathode. The external resistance was set at 1000 ohms.

Single-chamber batch MFCs were established using 250 mL Wheaton bottles and maintained at an ambient temperature of 24 °C (Figure 1). The anodic component was fabricated from two carbon cloth sections (CCP-2M, Fuel Cell Earth LL, Woburn, MA, USA), each measuring 3 × 5 cm. The cathodic component, with a diameter of 2.5 cm, was crafted from carbon cloth (Type B) and subsequently treated with a PTFE coating and a 5% platinum catalyst, following the methodology outlined by Cheng et al. [32]. The distance between the two electrodes was approximately 5 cm. Each MFC was filled with 250 mL of primary effluent (7 mS·cm^−2^) sourced from a domestic wastewater treatment plant located in Lyon, France. A consistent feed of 1 g·L^−1^ sodium acetate was introduced every 2 days. The anode and cathode were linked to a generator and polarized at a direct current voltage of 0.5 V for an initial period of 4 days, termed as MFC4d. After this 4-day interval, the polarization in the MFC4d was terminated, and the external circuit was completed with a resistance of 1000 ohms. For comparative analysis, a standard MFC, devoid of the initial polarization and equipped with an external resistance of 1000 ohms, was designated as the control (CtrlMFC). Both CtrlMFC and MFC4d were initiated simultaneously to ensure consistent experimental conditions and comparability between the two setups. After the initial period of 4 days, voltage evolutions in both MFC configurations were diligently recorded at 30-min intervals over a span of 20 days, utilizing a Hewlett Packard 3456 A Digital Voltmeter (Hewlett-Packard Development Company, L.P., Spring, TX, USA) in conjunction with an Agilent 34970 A Data Acquisition/Switch Unit (Keysight, Santa Rosa, CA, USA).

### 2.2. Electrochemical Characterization 

Upon stabilization of electricity production, which was observed after 17 days, polarization curves for the MFCs were recorded using a variable external resistance and a voltmeter. The surface current was determined with the following Equation (1):

(1) $$I=\frac{U}{R}\cdot \frac{1}{S}$$

where *U* is the voltage of the cell, *R* is the external resistance, and *S* is the surface of the anode. The power density was calculated with the following Equation (2):

(2) P=UI

For electrochemical characterization, cyclic voltammetry (CV) of the anodes was conducted employing a Voltalab-40 potentiostat (Hach Lange, Ames, IA, USA) within a three-electrode system. This system comprised the working electrode (anode), a saturated Ag/AgCl reference electrode, and a platinum wire functioning as the counter electrode. The potential range for the CV measurements spanned from −0.8 V to 1 V relative to Ag/AgCl, with a scan rate set at 10 mV·s^−1^.

### 2.3. DNA Extraction and Sequencing

Following a 17-day period, two anode sections, each measuring 1 cm, were excised and immersed in 1 mL of a 0.8% sterile NaCl solution, accompanied by a sterile ceramic bead sourced from MP Biomedicals, Illkirch, France. These anode sections underwent mechanical disruption using a FastPrep machine (MP Biomedicals) set to a velocity of 6 m.s^−1^ for a duration of 20 s. From the resultant suspension, 500 µL was allocated for total DNA extraction, employing a modified Genomic DNA from Tissue kit (Macherey-Nagel GmbH and Co. KG, Duren, Germany). The manufacturer’s instructions were followed except that the pre-lysis incubation was performed by first incubating the centrifuged sample with 180 µL T1 and 25 µL of proteinase K for 5 min at room temperature. This was followed by vortexing and a subsequent 10-min incubation at 70 °C. The enzymatic reaction was terminated by heating the samples to 95 °C for 5 min. The extracted metagenomic DNA underwent sequencing via the 454 technology. Additionally, amplification of the V3-V4 region of the 16S rRNA gene (*rrs*) was achieved using the forward primer 5′-TCGTCGG-CAGCGTCAGATGTGTATAAGAGACAGCCTACGGGNGGCWGCAG-3′ and the reverse primer 5′-GTCTCGTGGGCTCGGAGATGTGTATAAGAGACAGGACTACHVGGGTATCTAATCC-3′ (yielding an amplicon size of 460 bp). The amplified 16S ribosomal RNA gene (*rrs*) amplicons were subsequently sequenced utilizing the Illumina MiSeq System.

### 2.4. Proteins Extraction and Sequencing

Cellular pellets were subjected to lysis, denaturation, and reduction in a 6 M guanidine solution supplemented with 10 mM DTT in a 50 mM Tris buffer (pH 7.6). This mixture was incubated with agitation overnight at 37 °C. Subsequently, the solution underwent a 6-fold dilution using a 50 mM Tris buffer containing 10 mM CaCl_2_ (pH 7.6). Proteins present were enzymatically cleaved into peptides using sequencing grade trypsin (Promega, Madison, WI, USA) at a ratio of 1/100 (*wt*/*wt*). Insoluble cellular debris was eliminated via centrifugation at 2000× *g* for 10 min. Resultant peptides were desalted using C18 solid phase extraction (Waters, Milford, MA, USA), concentrated, filtered, and aliquoted, following the protocol delineated by Verberkmoes et al. [33]. Two-dimensional nano-LC MS/MS analyses of the samples were executed on an LTQ-Orbitrap hybrid mass spectrometer (Thermo Fisher, San Jose, CA, USA), as detailed in the literature [34]. Briefly, tryptic peptides underwent chromatographic separation over 22 h with escalating pulses (0–500 mM) of ammonium acetate, followed by a 2 h transition from aqueous to organic solvent. The LTQ operated in a data-dependent mode, encompassing: MS/MS on the top five ions identified in the full scan, dual microscans for both full and MS/MS scans, centroid data acquisition for all scans, and a dynamic exclusion parameter set to 1. 

Protein quantification was expressed in terms of the normalized spectral abundance factor (NSAF). NSAF calculations are grounded on spectral counts associated with peptides of a specific protein. A correction factor is applied to account for the higher probability of detecting a longer protein (due to it yielding more peptides) and is normalized to total spectral count for the run.

### 2.5. Sequence Analyses

#### 2.5.1. 16S Ribosomal RNA Gene Amplicon Sequence Analysis 

Sequences were joined using Pandaseq [35] ensuring a minimum overlap of 50 bp and a resultant sequence length ranging from 500 to 600 bp. Operational taxonomic units (OTUs) were delineated at a 97% identity threshold using QIIME [36] via the pick closed reference.py script. Taxonomy was assigned referencing the Greengene database. To ensure uniformity, all samples were normalized to the sequence count of the smallest sample. The Shannon index was computed at a pseudo-genus level using Equation (3):

(3) $$H^{\prime }=-\sum p_{i}\cdot ln(p_{i})$$

where *p_i_* is the proportion of the OTUi considered to belong exclusively to the annotated genus. The relative abundance of the major genus present in the samples was analyzed.

Based on the research conducted by C. Koch et al., which cataloged 69 species with the capability for anodic electron transfer, belonging to 37 genera [37], bacterial genera considered as potential EAB in this study were *Geobacter*, *Arcobacter*, *Desulfovibrio*, *Clostridium*, *Pseudomonas*, *Shewanella*, *Streptomyces*, *Bacillus*, *Aeromonas*, *Rhodoferax*, and *Escherichia*.

#### 2.5.2. Metagenomic and Metaproteomic Sequence Analysis

Metagenomic DNA sequences were annotated against the non-redundant protein (nr) database from NCBI using BLASTx facilitated by Diamond software (v2.1.8). For metaproteomic analysis, only proteins correlating with metagenomic sequences from the MFCs were retained, a process executed using R software (v3.5.3) and the seqinr package. Metaproteomic sequences were annotated against the specific MFC database using BLASTp via Diamond software [37], retaining only matches with an identity exceeding 40%. The most optimal assignments were retained for each sequence, and the NSAF values were aggregated for each assignment. Data interpretation was conducted using Megan software (v6.13.1) [11] and STAMP (v2.1.3) [38]. The gi map facilitated taxonomic structure analysis, while the SEED classification with the RefSeq map was employed for functional gene analysis. To discern the relative abundance of genes and proteins integral to intracellular and extracellular electron transfer, datasets were BLASTed against specialized functional databases. These databases, curated from the Bacteria RefSeq database of NCBI using keyword searches, encompassed proteins linked to functions such as cytochrome, hydrogenase, shuttles, acetate, and adhesion. These databases are accessible at genomenviron.org (accessed on 1 April 2018). Matches with an identity above 40% were retained, and the optimal assignment was selected for each sequence. Subsequently, the sequence count and relative abundance for each assignment were computed. To evaluate functional diversity, a Shannon index was calculated using the RefSeq protein identifier as the unit for both metagenomic and metaproteomic data.

## 3. Results

### 3.1. Effect of the Applied Voltage on Electricity Production

**Figure 2:**
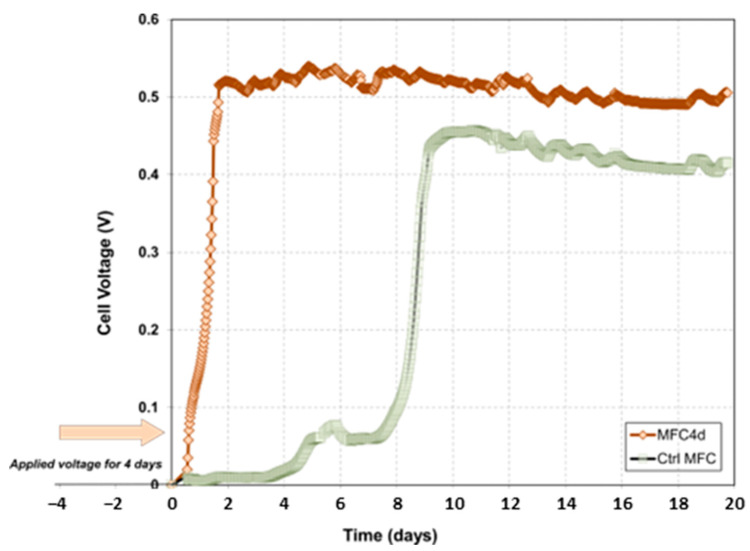
Evolution of the microbial fuel cell voltage as a function of time after the applied voltage (MFC4d) and without preceding phase of applied voltage (CtrlMFC). The external resistance was set at 1000 Ohms.

When the applied voltage in MFC4d was stopped, the exponential phase of electrical current production started within a day for a total of 4.5 days (Figure 2). In contrast, the control MFC (CtrlMFC) started its exponential phase 8 days after start-up. Notably, upon reaching its stationary phase, MFC4d exhibited a voltage that was approximately 50% higher (ranging between 450 and 500 mV) than the CtrlMFC, which peaked at approximately 450 mV. The application of voltage for a 4-day period appeared to reduce the latency phase and increase the stationary phase voltage. The peak power outputs for MFC4d and CtrlMFC were recorded as 38.2 mW·m^−2^ and 29.5 mW·m^−2^, respectively, with current densities of 150.0 mA·m^−2^ for MFC4d and 134 mA·m^−2^ for CtrlMFC. It is important to note that the power density of MFC4d was approximately 30% higher compared to CtrlMFC, demonstrating a significant difference in the electrical performance between the two setups. The difference in electrical performance between MFC4d and CtrlMFC can be attributed to the variation in catalytic activities of the anodic biofilms. This variation leads to activation overpotentials when current is drawn from the MFCs. When measured under no-current conditions, the open circuit voltages were nearly identical for both setups, registering 545 mV for MFC4d and 554 mV for CtrlMFC (Supplementary Materials Figure S1). Despite the elevated voltage observed in MFC4d, its maximum power density remained comparable to that of CtrlMFC. Cyclic voltammetry analyses of both MFCs had a single oxidation peak (Figure S2). This peak was detected at 300 mV vs. Ag/AgCl for MFC4d and 450 mV vs. Ag/AgCl for CtrlMFC. This difference might account for the observed voltage disparity between the two MFC configurations.

### 3.2. Bacterial Composition of Biofilms

**Figure 3:**
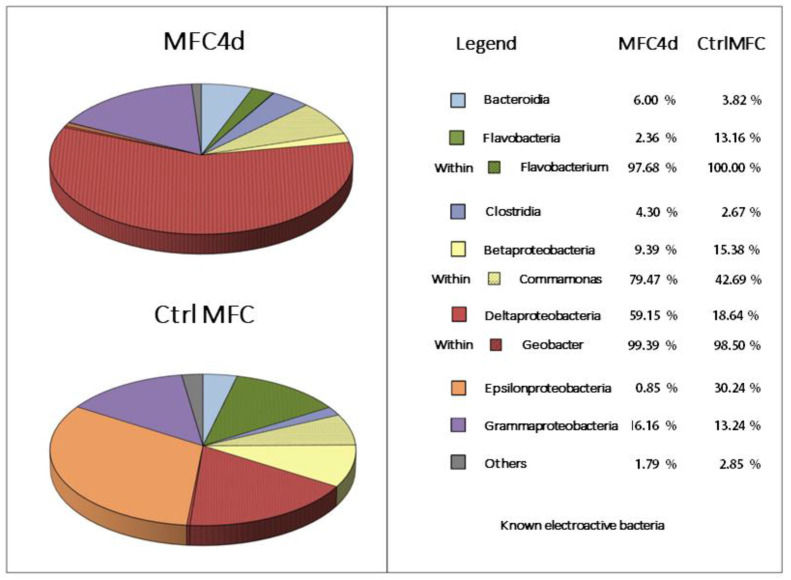
Bacterial community structure. The relative abundance of major classes and genus are represented for CtrlMFC and MFC4d samples.

Sequence analysis of the V3–V4 region of the 16S ribosomal gene (*rrs*) was conducted to determine the bacterial community structure (Figure 3). For the CtrlMFC, 122,834 sequences were obtained (average sequence length: 457.07 ± 11.14 bp) with 59.18% successfully annotated. In contrast, the MFC4d yielded 52,901 sequences (average sequence length: 463.30 ± 6.55 bp) with an annotation rate of 59.61%. The observed operational taxonomic units (OTUs) numbered 289 for CtrlMFC and 316 for MFC4d. Notably, the Shannon diversity index was higher in CtrlMFC (2.38) compared to MFC4d (1.84). In the CtrlMFC, the predominant taxa were Epsilonproteobacteria (30.24%), which were exclusively represented by *Arcobacter*; Deltaproteobacteria (18.64%), with *Geobacter* accounting for 98.50%; followed by Betaproteobacteria (15.38%), Gammaproteobacteria (13.24%), and Flavobacteria (13.16%). Other minor taxa included Bacteroidia (3.82%), Alphaproteobacteria (2.35%), and Clostridia (2.67%). In the MFC4d, the *Geobacter* genus was the most abundant and represented 58.86% of the community. Other significant taxa included Gammaproteobacteria (16.16%), Betaproteobacteria (9.39%), Bacteroidia (6.00%), and Clostridia (4.30%) with Flavobacteria constituting only 2.36%. The relative abundance of putative electroactive bacteria (EAB) was higher in MFC4d at 69.0% compared to 56.6% in CtrlMFC. While CtrlMFC exhibited a greater number of sequences and a higher Shannon diversity index, indicating a more diverse microbial community, MFC4d had a higher relative abundance of putative electroactive bacteria (EAB), particularly from the Geobacter genus. This suggests that the application of voltage in MFC4d favored the proliferation or attachment of *Geobacter* to the anode, aligning with its primary role in electricity generation. On the other hand, the CtrlMFC was dominated by Arcobacter, indicating a different microbial strategy for electricity production.

### 3.3. Comparison of Functional Gene Expression

**Figure 4:**
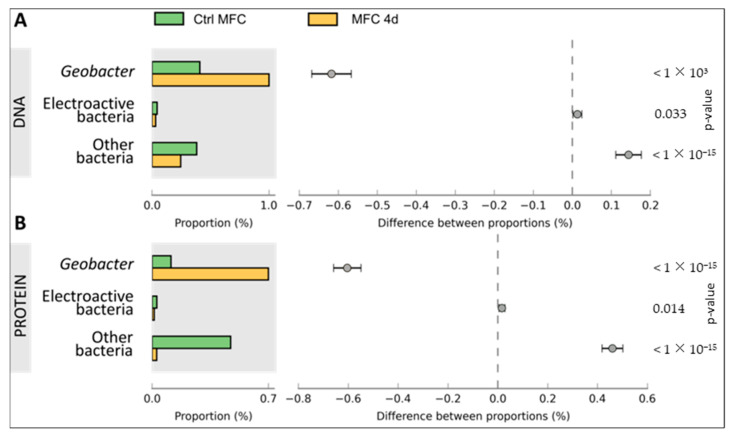
Cytochrome c oxidase function in microbial fuel cell communities. Proportion of sequences annotated as cytochrome c and difference between proportion of CtrlMFC and MFC4d in Panel (**A**) metagenomic data and Panel (**B**) metaproteomic data. Error bars are the 95% confidence intervals and were calculated with STAMP software.

**Figure 5:**
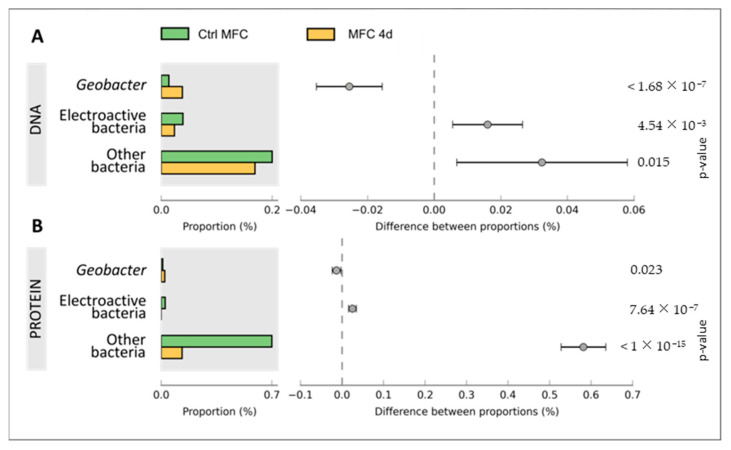
Acetate uptake function in microbial fuel cell communities. Proportion of sequences annotated as acetate uptake and difference between proportion of CtrlMFC and MFC4d in Panel (**A**) metagenomic data and Panel (**B**) metaproteomic data. Error bars are the 95% confidence intervals and were calculated with STAMP software.

**Figure 6:**
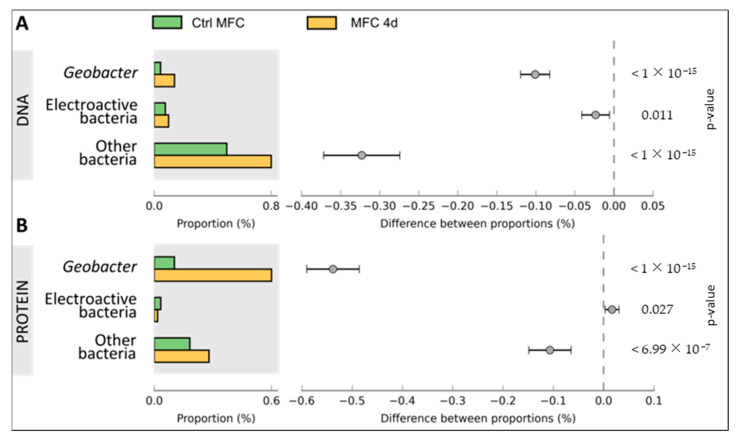
Hydrogenase function in microbial fuel cell communities. Proportion of sequences annotated as hydrogenase and difference between proportion of CtrlMFC and MFC4d in Panel (**A**) metagenomic data and Panel (**B**) metaproteomic data. Error bars are the 95% confidence intervals and were calculated with STAMP software.

Metagenomic analysis was conducted on DNA samples extracted from both CtrlMFC and MFC4d by utilizing 454 pyrosequencing. The resultant sequence counts were 251,718 for CtrlMFC (average sequence length: 239.79 ± 37.30 bp) and 201,041 for MFC4d (average sequence length: 239.42 ± 41.57 bp). Concurrently, metaproteomic analysis of protein extracts from both samples was performed using LC MS/MS. The CtrlMFC sample yielded 3443 unique peptide sequences (average sequence length: 77.08 ± 12.06 amino acids), while the MFC4d sample produced 2348 unique peptide sequences (average sequence length: 77.54 ± 13.22 amino acids) (Refer to Figure S3). Subsequent BLAST analysis of these metagenomic and metaproteomic sequences against the non-redundant protein database from NCBI was performed. The data were further processed and visualized using Megan and STAMP software v2.1.3 tools (See Figure S4 and Table S1). To home in on the electron transfer function, the sequences were also BLASTed against specialized databases with results presented in Figure 4, Figure 5 and Figure 6. Comprehensive data sets are accessible at www.genomenviron.org (accessed on 1 April 2018).

**Figure 7:**
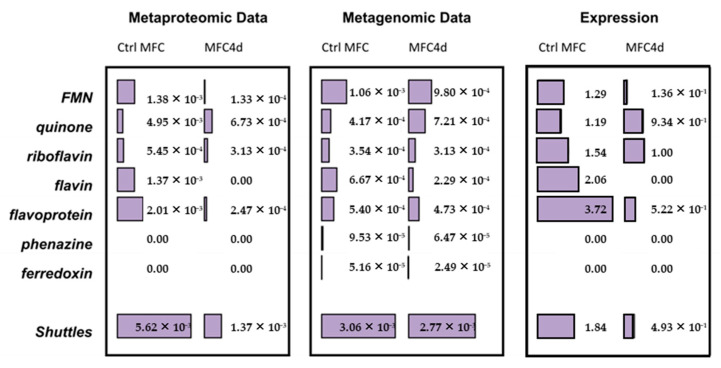
Shuttles Function. The relative abundance of functional gene and proteins is represented by the bar graph with the corresponding values for Ctrl MFC and MFC4d samples.

Hydrogenases and proteins associated with acetate and linked to electron donors and cytochrome c oxidase were the proteins considered to be related to direct electron transfer mechanisms. *Geobacter* proteins were more abundant in MFC4d than CtrlMFC for each of these functions. In addition, about 95% of cytochrome c oxidases identified in MFC4d were assigned to *Geobacter* (only 18% of cytochrome c oxidases in CtrlMFC were assigned to *Geobacter*). This result implicated *Geobacter* as a major player in the electron transfer in MFC4d. Overall acetate uptake proteins were lower in the MFC4d than in the CtrlMFC. Since the hydrogenase proteins were higher in the MFC4d, acetate might not have been as important a source of electrons as hydrogen for the dominant *Geobacter* in the MFC4d. To explore which bacteria might be involved in the electricity production in the CtrlMFC, the functions associated with other EAB were evaluated. EAB include members of the genera *Bacillus*, *Pseudomonas*, *Shewanella*, *Desulfovibrio*, *Aeromonas*, *Arcobacter*, *Streptomyces*, *Clostridium*, and *Rhodoferax*. Although there was three times more cytochrome c oxidase from EAB in the CtrlMFC than from those in the MFC4d, the relative abundance of these proteins was still relatively low. Since many EAB use shuttles to transfer their electrons to the anode, the relative abundance of proteins and genes associated with shuttle production was evaluated (Figure 7). The relative abundance of the genetic sequences associated with electron shuttle production were approximately the same for both MFCs. However, the relative abundance of proteins associated with electron transfer shuttles was four-times higher in the CtrlMFC than in the MFC4d (0.0056 and 0.0014, respectively). The relative abundance of proteins associated with FMN (FlavoMonoNucleotide) was also higher in the CtrlMFC than in the MFC4d (0.0014 and 0.0001, respectively). In the CtrlMFC, about 87% of these proteins were assigned to *Pseudomonas* and 13% to *Bacillus*. The proteins annotated as flavins were not detected in the MFC4d and their relative abundance was 0.0014 in the CtrlMFC. All flavins were assigned to *Pseudomonas* and identified as flavin reductases. Flavoproteins were the most abundant proteins in the CtrlMFC (0.020) and were assigned to *Streptomyces*. Finally, the relative abundance of proteins associated with riboflavins was 0.0050 in the CtrlMFC and 0.0003 in the MFC4d. In the CtrlMFC, riboflavin proteins were identified as riboflavin biosynthase and assigned to *Aeromonas*. Therefore, the electricity production in the CtrlMFC seemed to be the result of redox mediators belonging to a more diverse bacterial community than those in the MFC4d.

To evaluate the influence of the applied voltage on functional diversity and selection pressure, the Shannon index was calculated using the RefSeq protein identifier (Table 1). At the gene level, the Shannon indices for cytochrome c oxidase, hydrogenase, and acetate functions exhibited congruence. However, at the protein level, these functions demonstrated a reduced Shannon index in MFC4d compared to CtrlMFC. This suggests that the applied voltage in MFC4d favored the synthesis of more specialized proteins for these specific functions. Conversely, for the electron shuttle function, the Shannon index was elevated in CtrlMFC relative to MFC4d, both at the gene and protein levels. This indicates a distinct protein diversity profile between MFC4d and CtrlMFC (Figure S1).

## 4. Discussion

### 4.1. Electron Transfer Mechanisms in the Biofilms

Distinct electron transfer mechanisms were evident in the biofilms of both MFC setups. In MFC4d, the pronounced presence of *Geobacter* and its associated proteins, specifically annotated as cytochrome c, emphasized its pivotal role in electricity generation. *Geobacter*’s established mechanism of direct electron transfer through cytochrome c aligns with these results [9]. In addition, the diminished relative abundance of proteins linked to electron shuttles in MFC4d demonstrated a lesser reliance on this electron transfer mechanism. Hence, direct electron transfer likely predominated in MFC4d. While *Geobacter* is recognized for its nanowire production, the absence of the PilA protein, which is integral to nanowire formation, in both MFCs (refer to Figure S5) indicated a potential direct transfer from cytochrome c to the anode, bypassing nanowires. Conversely, in CtrlMFC, there was an elevated production of proteins associated with redox shuttles compared to MFC4d. Notably, the presence of FMN reductases and flavin reductases from *Pseudomonas* suggested a potential electron transfer mechanism via shuttles. Although *Pseudomonas* is documented as employing phenazines for electron transfer [39], these were not detected in the studied samples. Instead, *Pseudomonas* might predominantly utilize flavin family proteins for electron transfer in CtrlMFC. The identification of riboflavin biosynthase protein from *Aeromonas* in CtrlMFC further supported its potential involvement in electron transfer through the flavin family. While *Aeromonas* is recognized as an electroactive bacterium (EAB), its electron transfer mechanism remains speculative [8]. The role of *Streptomyces*, another potential EAB, in CtrlMFC is intriguing. Although flavoprotein reductases are part of its respiratory chain, their involvement in extracellular electron transfer remains unverified. Nevertheless, the protein analysis indicated that *Streptomyces*’ activity might reflect its potential contribution to electricity generation in CtrlMFC. In contrast to MFC4d, the CtrlMFC exhibited a limited presence of *Geobacter*-associated cytochrome c proteins. This, combined with the reduced expression of cytochrome and hydrogenase genes in CtrlMFC, implies a subdued activity of *Geobacter* in this setup. Interestingly, approximately 47% of cytochrome c proteins in CtrlMFC were attributed to *Acidovorax*, a bacterium identified in various anodic [40] and cathodic biofilms [41] and might be linked to its potential role in electron transfer.

### 4.2. Taxonomic and Functional Diversity in Microbial Biofilm

The influence of applied voltage on both taxonomic and functional diversity was assessed using the Shannon index, a metric encompassing richness and evenness. Previous studies have presented conflicting findings regarding the relationship between taxonomic diversity and power density in MFCs. Stratford et al. identified a positive correlation between taxa and power density and suggested that power density increased with increased taxonomic diversity [26]. Conversely, Sun et al. reported MFCs with elevated power densities exhibited reduced taxonomic diversity [26]. In the present investigation, the application of voltage for an initial 4-day period favored the proliferation of *Geobacter* within the anodic biofilm (59% in MFC4d and 18% in CtrlMFC), which subsequently reduced diversity. The taxonomic diversity was notably reduced in MFC4d, which underwent a voltage-assisted start-up phase (Shannon index values: 1.84 for MFC4d vs. 2.38 for CtrlMFC). This reduction was accompanied by a heightened operational electrical output (500 mV compared to 340 mV at 1000 ohms). Although both systems exhibited comparable maximum power densities, potential cathode limitations might be responsible. At the genetic level, functional diversities of cytochrome c oxidases, hydrogenases, and acetate functions were analogous between MFC4d and CtrlMFC. This similarity might be attributed to the significant presence of *Geobacter*, which is known for its diverse array of cytochrome c oxidases and hydrogenases [42,43]. For example, an analysis by Butler et al. of six *Geobacter* genomes described an average of 76 genes encoding cytochromes per genome. In contrast to gene-level functional diversity, protein diversity associated with cytochrome c oxidases, hydrogenases, and acetate functions was reduced in MFC4d. Furthermore, the functional diversity related to shuttle functions was more pronounced at both the gene and protein levels in CtrlMFC than in MFC4d. Although these functions are common in bacteria, they are absent in *Geobacter* genomes. Beyond their role in extracellular electron transfer, these functions participate in other cellular processes, including quorum sensing, which can modulate biofilm formation [44]. In conclusion, the initial application of voltage seemed to favor the selection of *Geobacter* and to promote the synthesis of specific proteins integral to extracellular electron transfer.

### 4.3. Effects of the Applied Voltage on Geobacter selection

**Figure 8:**
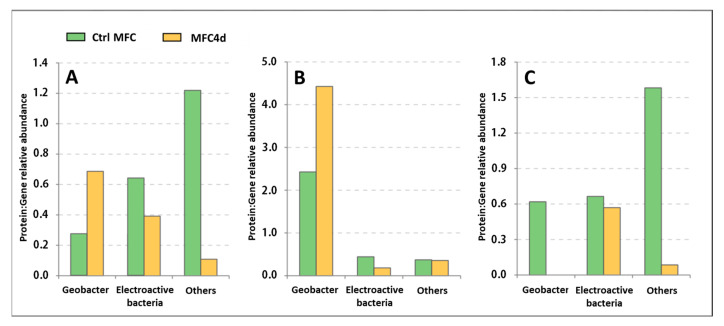
Ratio of relative abundance of proteins to genes for different functions (**A**) cytochrome oxidase, (**B**) hydrogenase, (**C**) acetate uptake.

The application of voltage between the anode and cathode during the colonization phase was observed to modulate the bacterial communities populating the anode. This not only changed the functional expression but also the electron transfer. In addition, the application of voltage hastened the start-up phase. A plausible hypothesis is that the applied voltage enhanced the anodic respiration activity of *Geobacter* due to the presence of an electromotive force during the initial phase. To determine the potential metabolic regulation differences in *Geobacter* between CtrlMFC and MFC4d, the ratio of the relative abundance of proteins to the relative abundance of the corresponding gene was calculated (Figure 8). The expression ratio for cytochrome c in MFC4d surpassed that in CtrlMFC, with values of 0.69 and 0.27, respectively. Similarly, the ratio for hydrogenase was elevated in MFC4d compared to CtrlMFC, with values of 4.43 and 2.43, respectively. Thus, the applied voltage appears to increase the expression of *Geobacter*’s electron transfer genes. Unlike other EAB, such as *Shewanella*, *Geobacter* has a limited range of soluble electron acceptors. During the initial phase for CtrlMFC, the absence of an electromotive force might have selected the pioneering bacteria on the anode that were capable of using soluble electron acceptors for growth. As the MFC voltage increased over time, the emergence of an electromotive force could have promoted anodic respiration in the control MFC and therefore accounting for the heightened bacterial diversity in CtrlMFC. This diverse community might exhibit a broader, yet less specialized, adaptation to extracellular electron transfer compared to *Geobacter*. Further investigations are needed to elucidate the influence of soluble electron acceptors on the development of anodic bacterial communities. In MFC4d, the presence of an electromotive force likely facilitated electron transfer to the anodic surface and mediated *Geobacter*’s earlier proliferation. *Geobacter*’s competitive edge over other EAB in MFC4d could be attributed to two factors. First, the selection of acetate as a substrate inherently favored by acetotrophic bacteria like *Geobacter*. Second, *Geobacter*’s potential superiority in employing direct electron transfer, which incurs fewer energetic losses during extracellular electron transfer compared to shuttle-mediated mechanisms, might confer a competitive advantage [45].

### 4.4. Advantages and Limits of Metaproteomic Analysis

Metaproteomic methodologies seemingly offer a more comprehensive assessment of microbial community activity compared to solely metagenomic strategies. This approach is employed to determine the functional roles of various microorganisms in ecosystems [31]. The LC MS/MS technique boasts superior sensitivity in detecting and identifying proteins compared to 2D-page analysis [46]. Nonetheless, the quantity of sequences identified through this method was considerably lower than those present in the metagenomic dataset and reduced the completeness of the comparative analysis between metagenomic and metaproteomic data. While this study utilized the comparison of protein relative abundance to gene relative abundance as an indicator of gene expression, this is not the same as direct measurement of gene expression. Furthermore, the protein annotation procedure is inherently more intricate than its metagenomic counterpart and renders generic metagenomic databases unsuitable for protein assignment. By annotating proteins based on the metagenomic dataset of the same samples as we did here, identification errors can be minimized. However, the accuracy of identification diminishes as the complexity of the environmental sample increases. Given that MFCs exhibit lower diversity compared to certain environments, such as soils, they present a useful environment to study functional expression and establish connections between function and taxonomy within microbial ecosystems. A significant challenge in exploring the functional expression of electron transfer in MFCs is the pervasive nature of the functions under investigation.

## 5. Conclusions

This study offers an in-depth exploration of the impact of applied voltage on the microbial community’s composition, functional diversity, and electron transfer mechanisms in anodic biofilms of MFCs. An important observation was the significant influence of applied voltage during the colonization phase in determining the biofilm’s taxonomic and functional attributes. Notably, the applied voltage promoted the prevalence of the Geobacter genus, renowned for its direct electron transfer capabilities, thereby augmenting the efficiency of electricity generation in MFCs. By integrating metaproteomic analyses, this research transcends the insights provided by metagenomic techniques alone, enabling a more granular comprehension of microbial community dynamics. This dual-method approach facilitated the identification of differentially expressed proteins pivotal to electron transfer processes and elucidated the functional contributions of diverse microbial members within the MFC ecosystem. The novelty of this work lies in its comprehensive approach to understanding the role of applied voltage in shaping microbial communities and its potential as a strategic intervention to modulate microbial community structure and function in anodic and cathodic biofilms of MFCs. In the future, it is imperative to further investigate the complex interactions among microbial species, their metabolic pathways, and external determinants that modulate their performance in MFCs. Such efforts will be instrumental in refining and optimizing bioelectrochemical systems for enhanced efficiency and broader applications. While this study highlighted the dominance of *Geobacter* under applied voltage, understanding the synergistic or antagonistic relationships between Geobacter and other microbial taxa could shed light on how these interactions influence overall system efficiency. The integration of other omics approaches, such as metabolomics and transcriptomics, alongside metaproteomics could offer a more holistic view of microbial activity. This would not only reveal the proteins being expressed but also provide insights into metabolic products and gene expression patterns. Furthermore, exploring the effects of varying voltage magnitudes, pulse frequencies, and durations might pave the way for determining optimal conditions for maximum electricity production and waste treatment efficiency. Lastly, transitioning from laboratory settings to real-world applications, such as wastewater treatment plants or industrial effluents, remains a pivotal step in harnessing the full potential of bioelectrochemical systems.

## Figures and Tables

**Table 1:** Functional diversity. Shannon index calculated with individual enzyme for Ctrl MFC and MFC4d samples and for metaproteomic and metagenomic data.

Functions	Metaproteomic Data		Metagenomic Data	
	Crtl MFC	MFC 4d	Crtl MFC	MFC 4d
Cytochrome c oxidase	2.575	1.602	5.836	5.277
Hydrogenase	2.457	1.944	5.517	5.439
Function associated to acetate	2.862	1.661	4.613	4.477
Shuttles	3.310	2.656	6.125	5.453

## Data Availability

The data presented in this study are available in Supplementary Materials.

## Supplementary Materials

The following supporting information can be downloaded at: https://www.mdpi.com/article/10.3390/microorganisms11112695/s1: Figure S1: Polarization curves and power density; Figure S2: Cyclic Voltammetry curves. CVs of MFC4d and CtrlMFC; Figure S3: Normalized spectral abundance factor (NSAF) for each protein sequence in A: MFC4d and B: CtrlMFC; Figure S4: Functional Proteins Structure. The relative abundance of proteins and the difference between proportion are represented for Ctrl MFC and MFC4d samples in function of SEED classification. The error bar are the 95% confidence interval and were calculated with STAMP software; Figure S5: Pili function in microbial fuel cell communities. Proportion of sequences annotated as pili and difference between proportion of CtrlMFC and MFC4d in A: metagenomic data and B: metaproteomic data. Error bars are the 95% confidence interval and were calculated with STAMP software; Figure S6: Cytochrome function in microbial fuel cell communities. Proportion of sequences annotated as cytochrome c and difference between proportion of CtrlMFC and MFC4d in A: metagenomic data and B: metaproteomic data. Error bars are the 95% confidence interval and were calculated with STAMP software; Table S1: Functional Gene Structure. The relative abundance of functional gene and the difference between proportion are represented for Ctrl MFC and MFC4d samples in function of SEED classification. The error bars are the 95% confidence interval and were calculated with STAMP software.
