# Supplementary material for: Metaproteomic and Metagenomic-Coupled Approach to Investigate Microbial Response to Electrochemical Conditions in Microbial Fuel Cells
Source: Microorganisms. 2023 Nov 3;11(11):2695. doi: 10.3390/microorganisms11112695 (PMC10673480; doi:10.3390/microorganisms11112695)
Supplement: Supplementary file 1 [file microorganisms-11-02695-s001.zip › microorganisms-2670518-supplementary.pdf]

Article

Metaproteomic and Metagenomic-Coupled Approach to Investigate Microbial Response to Electrochemical Conditions in Microbial Fuel Cells

Alexiane Godain [1,2], Timothy M. Vogel [2], Jean-Michel Monnier [1], Agathe Paitier [1,2] and Naoufel Haddour [1,*]

[1] Ecole Centrale de Lyon, INSA Lyon, University Lyon, Université Claude Bernard Lyon 1, CNRS, Ampère, UMR5005, 69130 Ecully, France
[2] Laboratoire d'Ecologie Microbienne, Universite Claude Bernard Lyon 1, UMR CNRS 5557, UMR INRAE 1418, VetAgro Sup, 69622 Villeurbanne, France
* Correspondence: naoufel.haddour@ec-lyon.fr; Tel.: +33-4-72-18-61-12

Keywords: Microbial Fuel Cell; Metagenomic; Metaproteomic; Electroactive bacteria; Extracellular electron transfer.

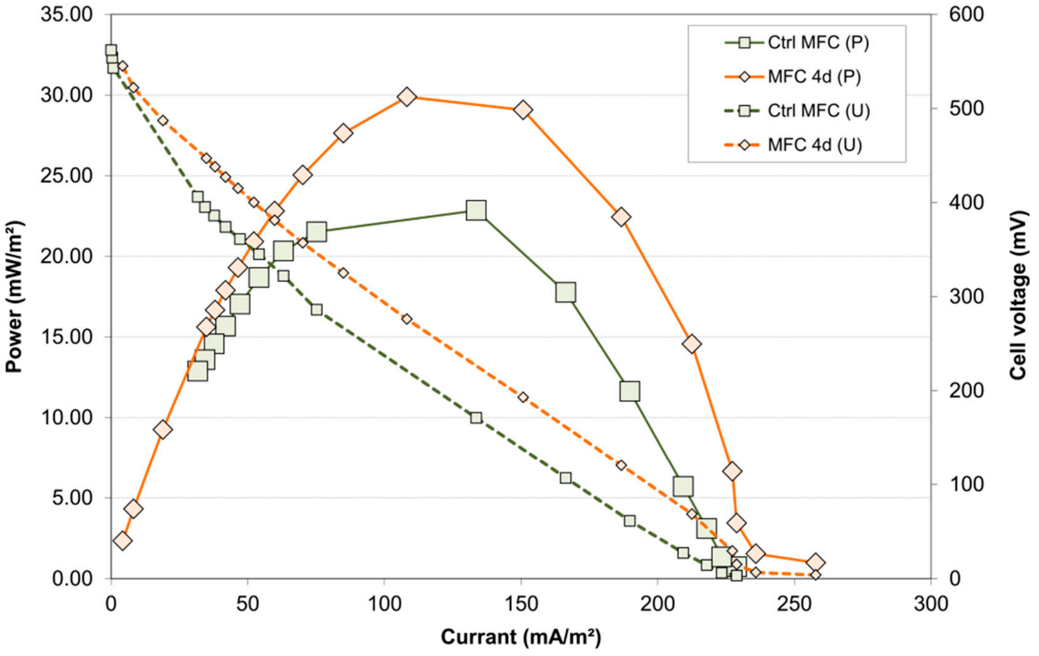

Figure S1. Polarization curves and power density.

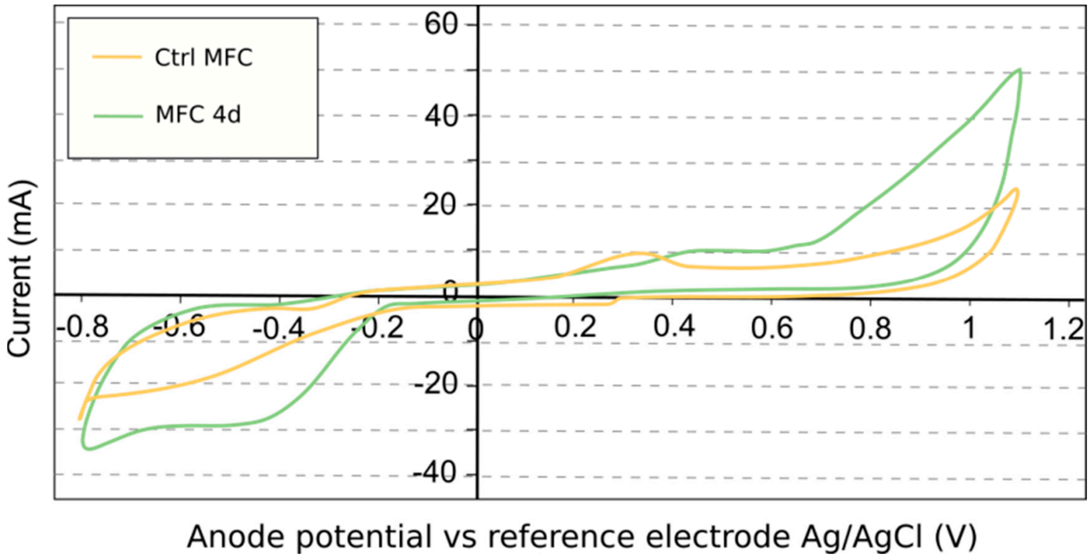

Figure S2. Cyclic Voltametry curves. CVs of MFC4d and Ctrl MFC

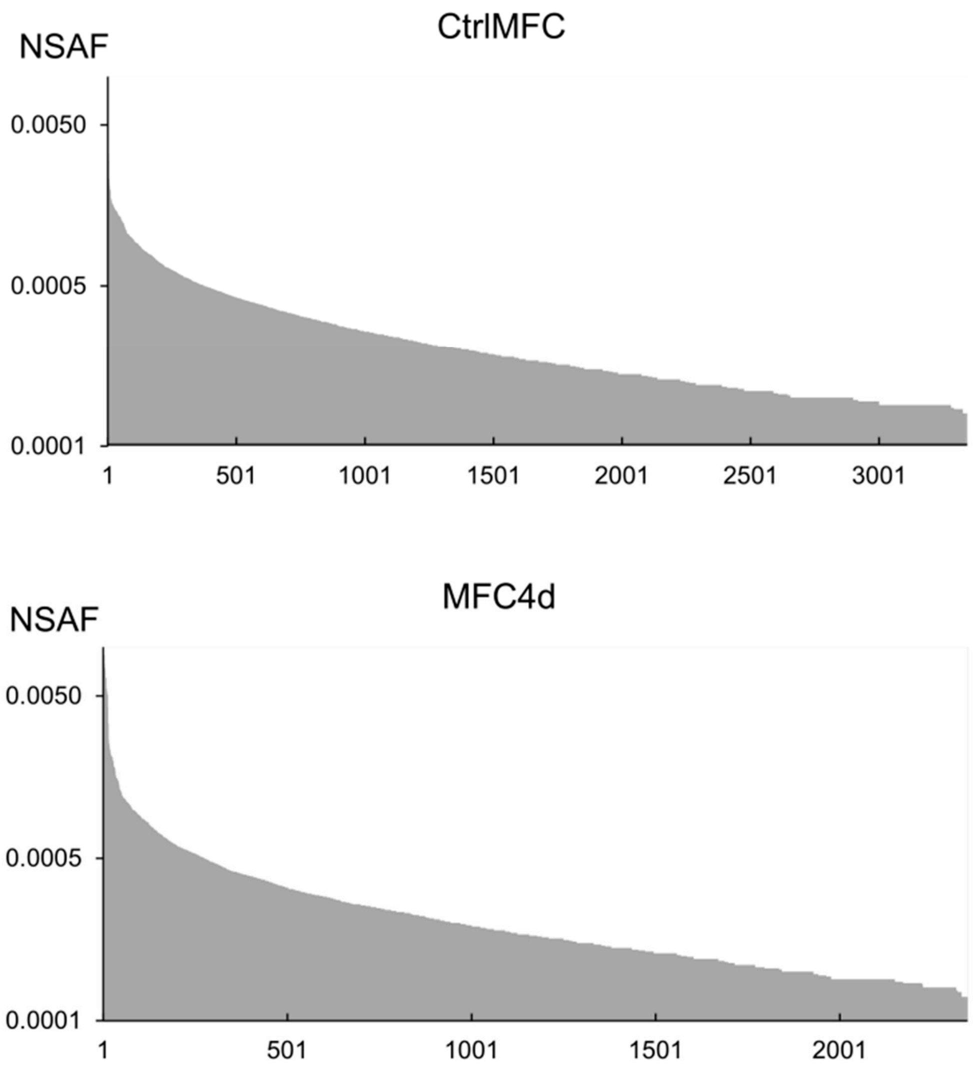

Figure S3. Normalized spectral abundance factor (NSAF) for each protein sequence in A: MFC4d and B: CtrlMFC

		Samples		non_redundant		acetate	
		nb_seq	filtred	nb_seq _db	%seq	nb_seq _db	%seq
DNA	Ctrl MFC	251718	-	96063464	82.48%	12168	0.26%
	MFC 4d	201041	-	96063464	77.49%	12168	0.24%
Protein	Ctrl MFC	112308	3522	96063464	99.36%	198	0.76%
	MFC 4d	104383	3232	96063464	97.74%	161	0.16%

		hydrogenase		cytochrome		pili	
		nb_seq _db	%seq	nb_seq _db	%seq	nb_seq _db	%seq
DNA	Ctrl MFC	60977	2.08%	351535	5.96%	115382	3.20%
	MFC 4d	60977	2.64%	351535	5.90%	115382	3.10%
Protein	Ctrl MFC	1001	0.35%	3953	1.33%	1543	0.32%
	MFC 4d	954	0.98%	2649	1.48%	1159	0.24%

Table S1. Functional Gene Structure. The relative abundance of functional gene and the difference between proportion are represented for Ctrl MFC and MFC4d samples in function of SEED classification. The error bar are the 95 % confidence interval and were calculated with STAMP software.

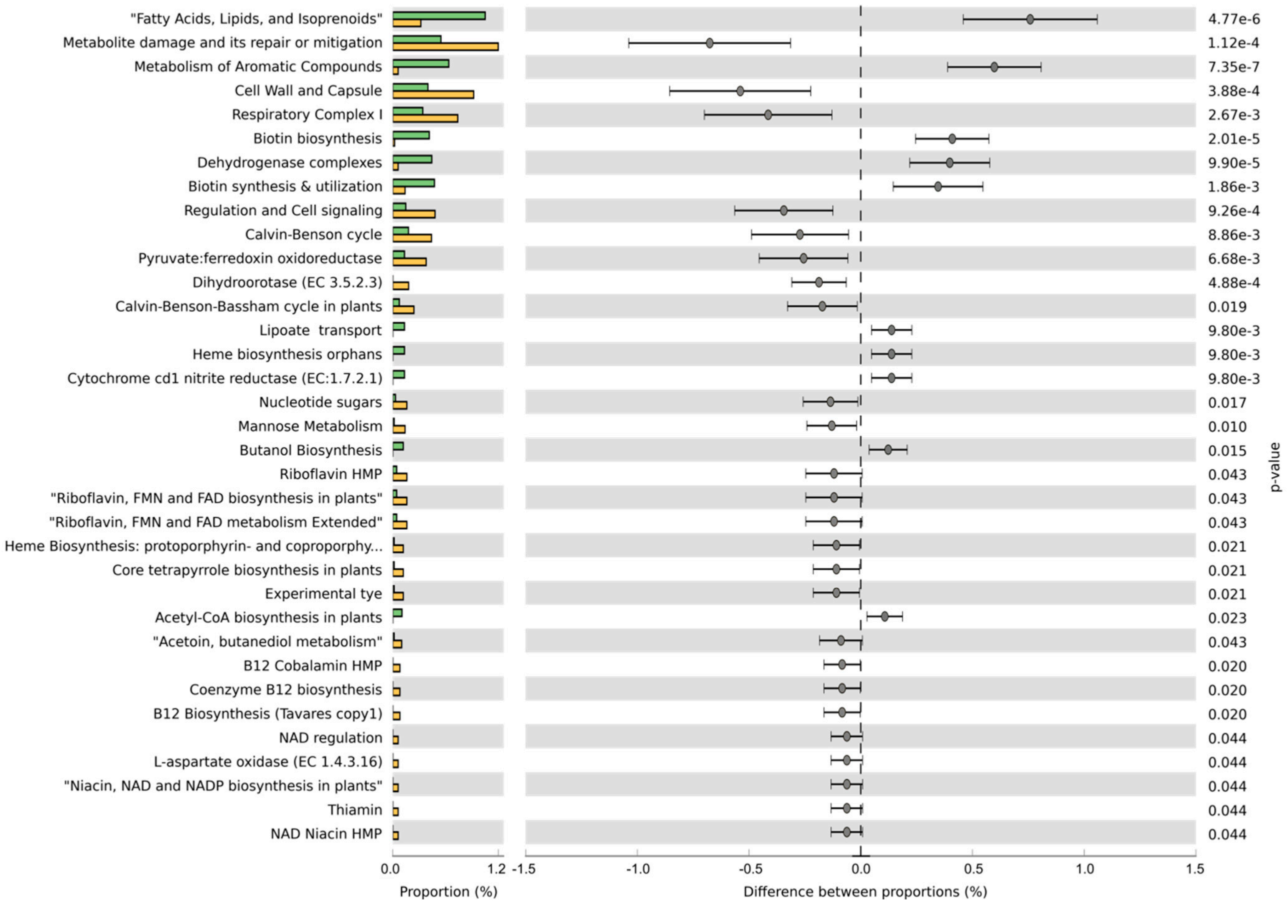

Figure S4. Functional Proteins Structure. The relative abundance of proteins and the difference between proportion are represented for Ctrl MFC and MFC4d samples in function of SEED classification. The error bar are the 95 % confidence interval and were calculated with STAMP software.

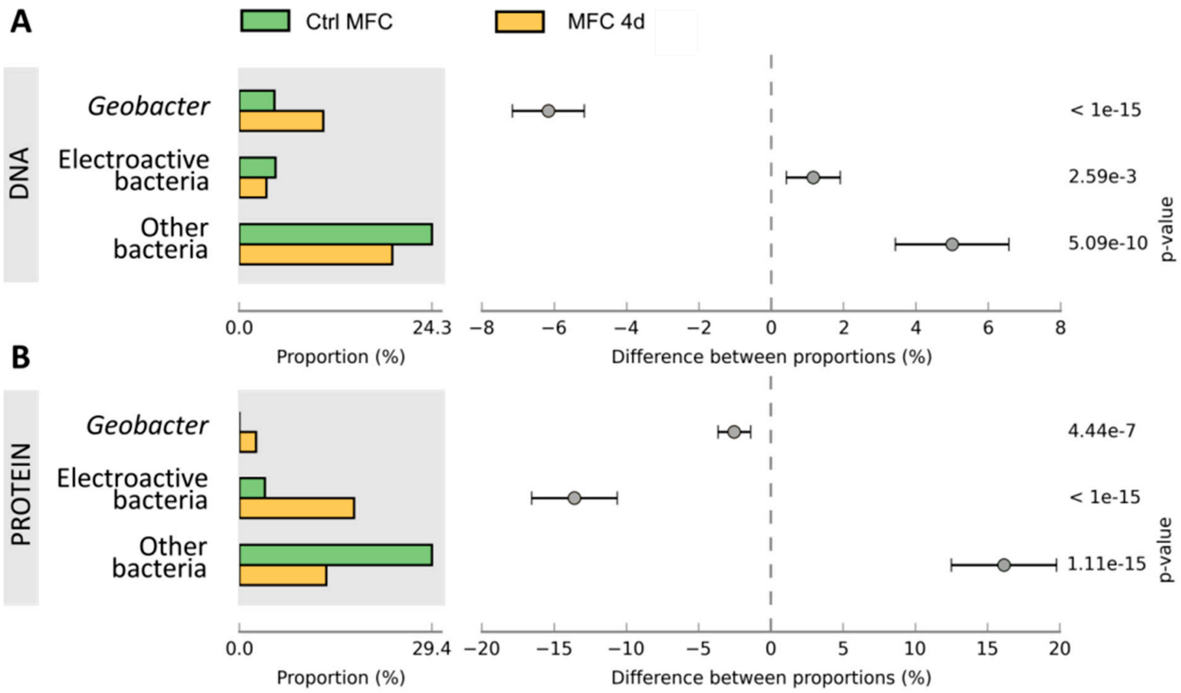

Figure S5. Pili function in microbial fuel cell communities. Proportion of sequences annotated as pili and difference between proportion of CtrlMFC and MFC4d in A:metagenomic data and B:metaproteomic data. Error bars are the 95 % confidence interval and were calculated with STAMP software.

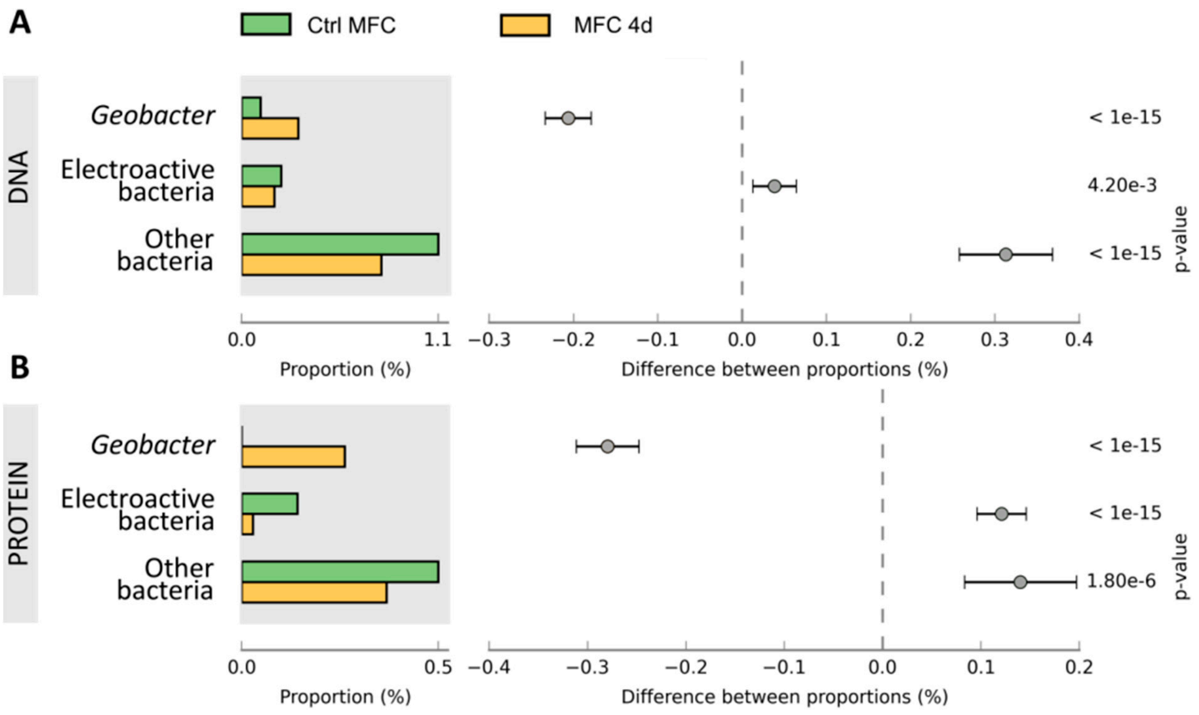

Figure S6. Cytochrome function in microbial fuel cell communities. Proportion of sequences annotated as cytochrome c and difference between proportion of CtrlMFC and MFC4d in A:metagenomic data and B:metaproteomic data. Error bars are the 95 % confidence interval and were calculated with STAMP software.
